# Looking at the Molecular Target of NS5A Inhibitors throughout a Population Highly Affected with Hepatitis C Virus

**DOI:** 10.3390/pathogens12060754

**Published:** 2023-05-24

**Authors:** Diogo Ramos, Miguel Pinto, Rodrigo Sousa Coutinho, Carolina Silva, Miriam Quina, João Paulo Gomes, Elizabeth Pádua

**Affiliations:** 1 Reference Laboratory of HIV and Hepatitis B and C, Department of Infectious Diseases, National Institute of Health, Av. Padre Cruz, P-1649-016 Lisbon, Portugal; diogo.ramos@insa.min-saude.pt (D.R.);; 2 Genomics and Bioinformatics Unit, Department of Infectious Diseases, National Institute of Health, Av. Padre Cruz, P-1649-016 Lisbon, Portugal; miguel.pinto@insa.min-saude.pt (M.P.); j.paulo.gomes@insa.min-saude.pt (J.P.G.); 3 Association Ares do Pinhal, Association for the Rehabilitation of Drug Addicts, Low-Threshold Methadone Substitution Program, R. José Inácio Andrade, Loja 2–A6–10B Quinta do Lavrado, P-1900-418 Lisbon, Portugal

**Keywords:** HCV, NS5A, resistance-associated substitutions, Sanger, NGS, drug users, Portugal

## Abstract

Hepatitis C virus (HCV) is associated with liver damage and an increased progression rate to cirrhosis and hepatocellular carcinoma. In Portugal, it is prevalent in vulnerable populations such as injection drug users (IDU). HCV is characterized by a high intra-host variability, and the selecting driving forces could select variants containing resistance-associated substitutions (RAS) that reduce treatment effectiveness. The main goal of this study was to analyze the sequence variation of NS5A in treatment-naïve IDU. The epidemiological and clinical status of hepatitis C were analyzed, and samples were sequenced by Sanger and Next-Generation sequencing (NGS) to assess RAS and confirm HCV subtypes. Phylogenetic classification was concordant: 1a, 52.4%; 1b, 10.7%; 3a, 20.2%; 4a, 8.3%; 4d, 7.1%; and one 2k/1b recombinant. A 1a/3a mixed infection was detected by NGS. RAS were found in 34.5% (29/84) of samples using Sanger sequencing, while in 42.9% (36/84) using NGS. In sequences from subtypes 1a and 1b, RAS K24R, M28V, Q30H/R, H58D/P/Q/R, and RAS L31M and P58S were detected, respectively. In subtype 3a, RAS A30S/T, Y93H and polymorphisms in position 62 were identified. Additionally, RAS P58L was detected in genotype 4. The strategy used for the molecular survey of baseline HCV resistance is of particular importance to achieve treatment effectiveness and contribute to the elimination of hepatitis C.

## 1. Introduction

Hepatitis C virus (HCV) is a blood-borne virus responsible for hepatitis C—a liver disease with a high chronicity rate. In the absence of treatment, persistent inflammation of the liver can progress to fibrosis, cirrhosis, or hepatocellular carcinoma [1]. In 2017, the World Health Organization reported that an estimated 71 million people were chronically infected worldwide [2]. Events from the recent past, including medical treatments with non-sterile tools (serial vaccination), blood transfusions, and the sharing of materials regarding drug abuse, led to the infection spreading during the last century [3], highly affecting drug addicts and injecting drug users (IDU).

HCV is an extremely dynamic virus characterized by its high replication rates and genetic variability, which affects treatment and the development of a vaccine against the virus [1,4]. Identification of HCV variants circulating worldwide have increased in recent years [5]. Still, during the last decade, the development of new drugs against HCV allowed the use of multiple direct acting antivirals (DAA) in the therapeutic regimens for infected patients [6]. These treatment options have sustained virological response (SVR) rates reaching 95.0% and generally include two or more DAA that target specific non-structural genes of the virus (NS3/4A, NS5A, and NS5B), inhibiting their function [1].

Despite the success of the DAAs, some issues have attracted scientific interest, such as the detection of resistance-associated substitutions (RAS), which are found in specific positions in the amino acid sequences of HCV viral proteins NS3, NS5A, and NS5B—yielding resistant variants of the virus [1]. These viral proteins present highly intrinsically disordered domains with a complex conformational landscape based on interactions with its effectors, such as those described in the literature for the NS3 protein [7]. This fact makes it a challenge to find effective therapies against hepatitis C. RAS can be present at baseline, in infected patients who have never experienced treatment (treatment- naïve), or in patients whose treatment has failed (treatment-emergent) [8].

The NS5A encoding gene is one of the most variable within the HCV genome, and variants characterized by the presence of mutations that generate RAS in its protein are very stable and can persist for an indefinite period of time [9]. In the literature, several RAS were described in NS5A, with some of them being detected in multiple genotypes/subtypes of HCV, such as L/M28S/T, P29S, Q/L30H, L31I/M/V, P32L/S, A/E92K, and Y93C/H/N/R/S [1,9].

Although methadone programs have been successfully implemented, Portugal is one of the European countries that has reported higher rates of hepatitis C infection in drug users, varying from 60.0% to 80.0% [10,11]. This population is especially vulnerable because of their risky behaviors and usually do not adhere to conventional healthcare facilities for the treatment of hepatitis C. In a study conducted on a population of drug users attending a low-threshold methadone program in Lisbon, the prevalence of HCV antibodies was 67.6%, and RNA viral load was detected in 68.4% of the enrolled individuals [12].

The main goal of this study was to perform a genetic analysis of the sequence variation of the viral target of NS5A inhibitors, mainly used in therapeutic regimens against HCV infection, by using Sanger and Next-Generation Sequencing (NGS) methodologies. This strategy allowed us to investigate the potential presence of mixed infections or genetic recombination based on previously available data from the NS5B viral region. Furthermore, an epidemiological and clinical characterization of hepatitis C in the chronically infected drug users enrolled in the study was also performed.

## 2. Materials and Methods

### 2.1. Specimens and Study Population

A global characterization of the population of drug addicts attending a Mobile Low-Threshold Methadone Program in Lisbon is available in a previous study [12]. The samples enrolled in the current study were collected between June 2015 and May 2016 and correspond to a subpopulation of randomly selected drug users with chronic hepatitis C. Plasmas were analyzed at National Institute of Health after obtaining informed consent from the participants. A total of 90 individuals who were naïve to treatment with DAA were enrolled in the current study (see Supplementary Table S1). All HCV genotypes in samples were previously described using NS5B region [12].

### 2.2. HCV RNA Extraction, cDNA Synthesis and PCR Amplification

HCV RNA was extracted and purified from 280 µL of plasma and eluted in 50 µL of elution buffer using the QIAamp Viral RNA mini kit (QIAGEN, Hilden, Germany). Complementary DNA (cDNA) was synthetized using the RT—kit plus (Nanogen Advanced Diagnostics, Buttigliera Alta, Italy) according to the manufacturer’s instructions. For the PCR reaction, the High-Fidelity PCR Master kit (Roche, Mannheim, Germany) was used with the primers, as described elsewhere [13]. Standard PCR reaction was performed in a volume of 50 µL per reaction, and between 10 µL and 15 µL of cDNA were submitted to amplification. The concentrations of the primers used were: NS5A-2F (forward) at 0.6 µM and NS5A-R and NS5A-3R at 0.3 µM. Thermocycling conditions started with an initial denaturation step of 94.0 °C for 2 min, followed by 10 cycles of 94.0 °C for 15 s, 48.0 °C for 70 s and 72.0 °C for 1 min, an additional 35 cycles of 94.0 °C for 15 s, 50.0 °C + 0.3 °C/cycle for 30 s and 72.0 °C for 1 min + 5 s/cycle, and a final extension step of 72.0 °C for 7 min. 

### 2.3. Products Detection, Purification and Sequencing Methods

Amplified PCR products were analyzed by electrophoresis through a 1.8% Agarose gel (SeaKem LE agarose-Lonza, Basel, Switzerland), and the fragments were purified using ExoSAP-IT (USB, Cleveland, OH, USA) according to the manufacturer’s instructions. Direct sequencing of NS4B-NS5A fragments by using Sanger method was performed starting from the purified products and using the BigDye Terminator Cycle Sequencing Ready Reaction kit (Applied Biosystems, Foster City, CA, USA) with the same primers as the PCR reaction. Capillary sequencing was performed in ABI Prism 3130xl Genetic Analyzer (Applied Biosystems, Foster City, CA, USA). NGS of NS4B-NS5A fragments was achieved by subjecting the amplicons to the Nextera XT protocol for preparation of NGS libraries and paired-end sequenced (2 × 150 bp) in the NextSeq 2000 instrument (Illumina, San Diego, CA, USA) according to the manufacturer instructions.

### 2.4. Analysis of Sequencing Results

Sequences obtained using Sanger sequencing were analyzed using Chromas v.2.6.6 to correct nucleotide misreads and then imported to BioEdit Sequence Alignment Editor v.7.2.5 [14] for alignment and construction of NS4B-NS5A consensus sequences—representative of each individual. For each nucleotide position, the nucleotide corresponding to the highest intensity peak was considered. In cases where two overlapping peaks were found, IUPAC nucleotide code was used to describe the degeneracy of the consensus regions.

NGS sequence read quality analysis and mapping was conducted using the bioinformatics pipeline implemented in the INSaFlu web platform (https://insaflu.insa.pt; https://github.com/INSaFLU, accessed on 20 June 2022) [15], which allows amplicon-based NGS data analysis. INSaFlu’s default parameters were considered for all analysis. Briefly, only variants present in the samples at frequencies greater than 51.0% were considered for the generation of NGS NS4B-NS5A consensus sequences. Mutations present at frequencies of 10.0%, 2.0%, and 1.0% (“minor variants”) were validated for sites with depth of coverage of at least 100-fold, 500-fold, and 1000-fold, respectively. To map the reads, a specific reference sequence for each genotype/subtype of HCV was uploaded: 1a (HQ850279.1), 1b (EU781828.1), 3a (X76918.1), 4a (DQ418789.1), and 4d (DQ418786.1). Only samples that presented a horizontal coverage above 90.0% and an average vertical coverage of 30 times were validated.

### 2.5. Phylogenetic Analysis

Reference sequences of HCV genotypes and subtypes circulating in Portugal were retrieved from Los Alamos HCV sequences database (http://hcv.lanl.gov, accessed on 29 December 2021). The accession numbers were: 1a (AF009606.1, EF407457.1, HQ850279.1), 1b (EU781827.1, EU781828.1), 2a (D00944.1, HQ639944.1), 2b (AB661382.1, AB661388.1), 3a (JN714194.1, X76918.1), 4a (DQ418789.1, DQ988074.1), 4c (FJ462436.1), 4d (DQ418786.1, EU392172.1), 5a (AF064490.1), 6a (EU246930.1), and 2k/1b (AY587845.1). NS4B-NS5A consensus sequences were aligned with the reference sequences, and two phylogenetic trees were generated—one for each sequencing method—in MEGA (Molecular Evolutionary Genetic Analysis) v.11.0.9 [16] using the neighbor-joining method [17] and Kimura 2-parameter model [18]. The strength and reliability of the three topology was evaluated with the bootstrap test [19] using 1000 replicates. Sequence clusters were considered significant with values greater than 70.0%.

### 2.6. Mutation Analysis

The presence of NS5A RAS was evaluated in sequences obtained from the two sequencing methods used (i.e., Sanger and NGS), thus allowing the comparison of results from the two techniques that have different detection limits. NS4B-NS5A consensus sequences obtained from Sanger sequencing were cleaved to retrieve only the region corresponding to the NS5A gene. Nucleotide sequences were then translated to amino acid sequences using BioEdit Sequence Alignment Editor v.7.2.5 [14]. On the other hand, identification of RAS in the sequences obtained by NGS relied on the analysis of the detailed reports provided by INSaFLU. Variants interpretation was conducted in BioEdit Sequence Alignment Editor v.7.2.5 [14] after deletion of NS4B fragment and translation into protein. Substitutions were examined, depending on the HCV genotype/subtype, in previously described positions 24, 26, 28, 29, 30, 31, 32, 38, 58, 62, 92, 93 of NS5A [1,9]. The reference sequences used were the same that were uploaded to map the reads from NGS.

### 2.7. Ethical Approval

The work conducted in the laboratory was completed using anonymized samples. Ethical approval was obtained by the Ethics Committee of the Portuguese National Institute of Health. All participants received information about the study by the team of social workers who supported the methadone program in mobile health units, and gave their freely informed consent.

## 3. Results

### 3.1. Population Characteristics 

Individuals were aged between 29 and 61 years (mean of 44 years), and the majority of them were Portuguese males (84.4%; 76/90). Regarding risk factors for developing more severe diseases, 35.6% (32/90) of the examined population claimed to have excessive alcohol consumption and 81.1% (73/90) declared the use of intravenous drugs. Of these, 45.2% (33/73) claimed to have shared materials related to injection-based drug use. For 79 individuals, we also collected data regarding the liver disease stage (obtained by transient liver elastography), which have the following distribution: 47.4% (37/78) at F0–F1 stage, 21.8% (17/78) at F2 stage, 14.1% (11/78) at F3 stage and 16.7% (13/78) at F4 stage.

### 3.2. HCV Genotyping and Phylogenetic Analysis

In the group of 90 samples under investigation, HCV NS4B-NS5A region was amplified in 93.3% (84/90), and all amplified products were successfully sequenced using Sanger and NGS methods. 

**Figure 1:**
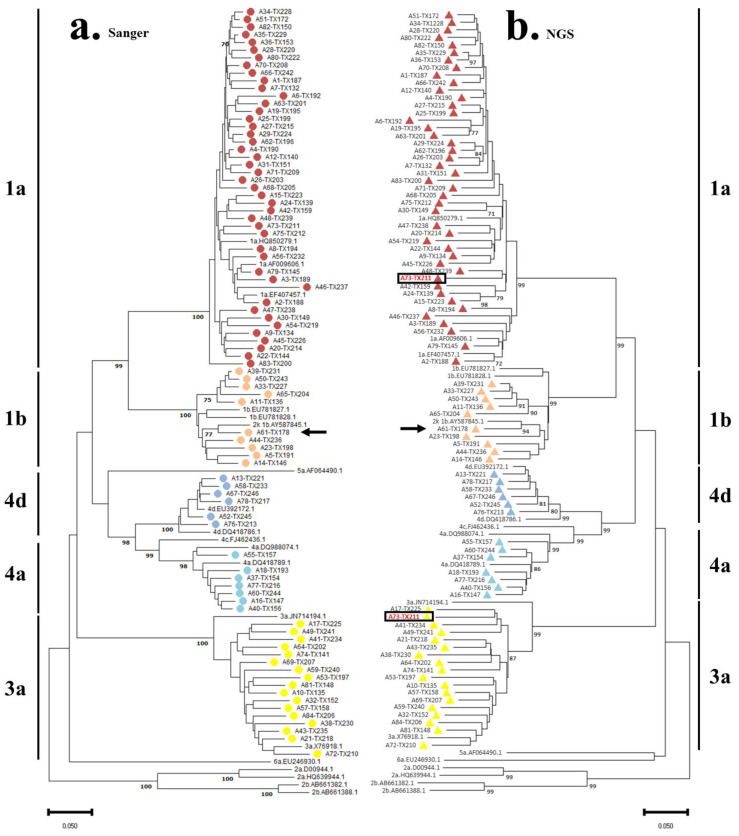
Phylogenetic neighbor-joining trees of the HCV sequences in the study as obtained by ● Sanger sequencing (**a**) and by ▲ NGS (**b**) based on the alignment of NS4B-NS5A genomic regions. The rectangles highlight the detected mixed infection; all positions containing gaps and missing data were eliminated from the analysis. Reference sequences are indicated by HCV subtype and GenBank accession number. The scale bar indicates the number of substitutions per site. Arrows indicate the 2k/1b recombinant.

Figure 1 shows the two neighbor-joining trees obtained from the analysis of the NS4B-NS5A sequences obtained by Sanger and NGS from all studied samples and reference sequences representative of HCV diversity in Portugal. The high robustness observed in both tree’s clusters, supported by the high bootstrap values obtained, allowed the classification of all samples in terms of HCV genotype and subtype. The results corroborate the previously known classification, using data available from the NS5B genomic region [12]. Overall, most samples were classified as HCV subtype 1a—corresponding to 52.4% (44/84). The only difference between the two trees laid in the cluster corresponding to HCV subtype 3a, where NGS allowed the detection of a mixed infection by this subtype and subtype 1a, was in sample A73-TX211 (cross-contamination was excluded due to the uniqueness of the subtype 3a sequence). Therefore, in the tree constructed using the sequences obtained from Sanger sequencing, 20.2% (17/84) of the samples were classified as belonging to subtype 3a. On the other hand, using NGS sequencing, 21.4% (18/84) of the samples were classified as belonging to this same subtype. The remaining samples were classified as follows: 10.7% (9/84) belonging to subtype 1b, 8.3% (7/84) belonging to subtype 4a, and 7.1% (6/84) belonging to subtype 4d. A recombinant 2k/1b was also identified (1.2%).

### 3.3. Analysis of Resistance-Associated Mutations

**Figure 2:**
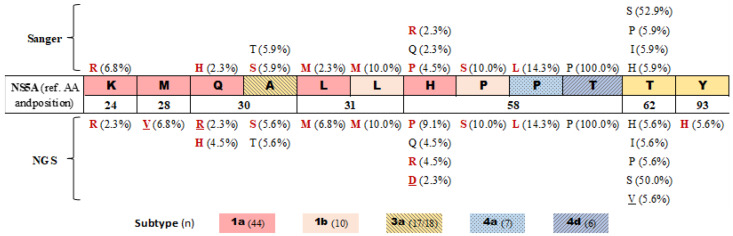
Amino acid substitutions detected in NS5A positions of interest by sequencing methods (Sanger at the top; NGS at the bottom) and proportions in the subpopulations corresponding to HCV subtypes. For NGS, proportions were determined considering the two sequences generated for sample A73-TX211 (mixed infection). Substitutions also identified by NGS are underlined, and those involved in resistance to NS5A inhibitors are highlighted in bold.

Resistance mutations to NS5A inhibitors were investigated for all 84 amplified samples, considering the results obtained from direct sequencing with Sanger and NGS. Figure 2 shows the substitutions detected in NS5A positions of interest and the proportions in the subpopulations corresponding to HCV subtypes. In all samples, the mean depth of coverage (90,987×; variation from 55,534× to 117,044×) obtained using NGS methodology allowed for the detection of amino acid substitutions in sequences present at frequencies of 1.0% or greater. An exception to this was sequence 3a from sample A73-TX211 (mixed infection), as its coverage (105×) only allowed the detection of substitutions at frequencies of 10.0% or greater. The mean depth of coverage per sample is available in Supplementary Table S1. Sanger sequencing allowed the identification of 32 amino acid substitutions—distributed by 29 consensus sequences (34.5%; 29/84). At the same time, NGS allowed the identification of 44 amino acid substitutions [16 in consensus sequences (>50.0%) and 28 as minor variants (≤50.0%)]—distributed by sequences of 36 samples (42.9%; 36/84). The vast majority of substitutions was detected in samples classified as belonging to HCV subtypes 1a and 3a. Substitution K24R was identified in three sequences (6.8%; 3/44) from subtype 1a using Sanger methodology. However, it was only detected in one sequence (2.3%; 1/44) from subtype 1a using NGS methodology. Substitution T62V, found in the subtype 3a sequence of the mixed infection sample A73-TX211, was detected with an NGS frequency of 88.7%. Other substitutions only identified using NGS were present in samples at frequencies ranging from 1.2% to 22.1% (mean = 6.0%). The complete data per sample regarding mutational analysis, including all amino acid substitutions detected using both sequencing methods, all combinations, and the frequencies in which substitutions were detected in NGS, are available in Supplementary Table S1.

## 4. Discussion

The emergence of HCV-resistant variants to DAAs is continuously increasing the need for HCV resistance testing to ensure the maximum efficacy of treatments and minimize virological relapses. The non-existence of standardized assays to study HCV RAS is limiting because it forces the use of in-house assays, meaning that most results cannot be compared due to the different performances of the methods [20]. Even though the reliability of techniques based on Sanger sequencing is very high, its sensitivity has been reported to vary from 10.0% to 25.0% [20]. In fact, deep sequencing methods allow a more sensitive analysis with the possibility to choose different cutoffs. Nowadays, there is some controversy about whether mutations present in samples at frequencies below 15.0% may be clinically relevant. However, some reports consider that baseline RAS using a 1.0% cutoff may become predominant in the viral quasispecies and result in lower SVR rates in comparison to SVR rates achieved in individuals without RAS after treatment with DAAs [21].

The results showed that the use of deep sequencing clearly has advantages in comparison with Sanger sequencing, such as the detection of RAS in viral variants present in frequencies lower than 25.0% (minority variants), the higher accuracy for the classification of subtypes, recombinant forms, and the detection of mixed infections by different subtypes of HCV in the sample of one infected patient [22]. Although these events are uncommon in the general population, they could occur in vulnerable groups such as high-risk IDU who share materials in injection-based drug abuse [23], and this may explain some virologic failures and, together with the presence of RAS, negatively impact the SVR rates obtained after treatment with the new interferon-free regimens [24].

The NGS results led to the identification of a mixed-infection by subtypes 1a and 3a in sample A73-TX211—a sample previously classified as HCV subtype 1a using Sanger sequencing. Although no information regarding alcohol abuse and liver state is available, this sample corresponds to one IDU that claims to have drug-injecting material-sharing behaviors, which agrees with the finding of a mixed infection with two HCV subtypes. Furthermore, in studies focused on vulnerable groups, mixed infections by these same subtypes were also identified, namely in prison inmates from Australia who identify as IDU [25] and in IDU from Germany and Russia [23].

The HCV recombinant form derived from sample A61-TX178 was observed in the phylogenetic trees grouped with the reference sequence RF1_2k/1b (accession number AY587845.1) and in the cluster of sequences of subtype 1b, which is expected because the crossover point described for this recombinant is located in the NS2 region of the viral genome [26]. A more detailed analysis of sample A61-TX178 was performed in SimPlot v.3.5.1 (https://sray.med.som.jhmi.edu/SCRoftware/SimPlot/, accessed 16 December 2022) with the sequence data from C/E1, NS3 and NS5B genomic regions available from a previous study [12]. These results are available in Supplementary Figure S1. It is important to note that RF1_ 2k/1b was already identified in a study conducted in 2013 in Portugal [27], which suggests that this recombinant, with its unknown clinical and/or therapeutic features, is in circulation in the country.

The most common subtypes that infect these IDU were 1a (52.4%) and 3a (20.2%-21.4%), which is in accordance with previous studies [12]. These subtypes are also frequent in infected IDU in several European countries [10]. Of note, in the studied population, 32 individuals reported that they excessively consume alcohol, which negatively impacts on liver diseases and favors the progression of HCV infections [28], and at least 24 individuals had advanced fibrosis or cirrhosis, making them more likely to fail treatment with DAAs. RAS in HCV NS5A protein are often detected in DAA-naïve patients, persist for many years, and are associated with lower SVR rates in specific groups of individuals, including those infected with HCV subtype 1a or genotype 3 and/or those with cirrhosis [20,29].

Overall, the results of the amino acid substitutions obtained from the two sequencing methods using the same level of sensitivity (10.0–25.0%) were compatible. One exception to this was RAS K24R, which was detected in three sequences (samples) of subtype 1a derived from Sanger (two of them with mixed peaks in the electropherograms) and detected only in one of these sequences of subtype 1a from NGS. These results highlight a possible problem that occurred in the Sanger sequencing process, which led to an incorrect reading of the nucleotides in the positions that codify this amino acid.

Most of the amino acid substitutions identified in this study were previously described as conferring certain levels of resistance against specific DAAs approved by the U.S. Food and Drug Administration and European Medicines Agency. Cross-resistance to the majority of RAS is frequently reported [8], making it difficult to describe all NS5A inhibitors affected by specific RAS. For example, RAS K24R, detected in sequences from subtype 1a, confers some level of resistance to Ledipasvir, Q30H,3 and L31M to Daclatasvir and Ledipasvir, and H58P/Q/R are secondary mutations presenting low levels of resistance to DAAs [8,30,31,32]. NGS enabled more detailed analysis with the detection of RAS M28V that confers resistance to Ombitasvir and Daclatasvir and Q30R and H58D, two high-level resistant RAS to Daclatasvir, Ledipasvir, and Ombitasvir [20,30,32]. In subtype 1b sequences, the two RAS identified were L31M and P58S, with the latter being a minor mutation that normally increases the resistance of primary mutations but does not confer resistance by itself [33]. Furthermore, in subtype 3a sequences, substitutions of A30S/T were detected, both having a minimal impact on the susceptibility to Daclatasvir in vitro [34]. The polymorphisms detected in amino acid position 62 seem to have little or no effect on Daclatasvir susceptibility in cell culture, although they might modify the effects of other NS5A substitutions [34]. NGS also allowed the detection of RAS Y93H, which is one of the most important RAS and is commonly detected in sequences from most HCV genotypes [9]. This RAS confers significant resistance to all NS5A inhibitors in vitro and reduces the SVR rates in patients infected with genotype 3 treated with Sofosbuvir/Velpatasvir [31]. Studies with infected patients whose treatment with Ledipasvir and Sofosbuvir plus ribavirin failed to detect the presence of RAS P58L [35], which was also detected in the current study in one sequence of genotype 4a. Lastly, for HCV subtype 4d, no RAS were identified. However, in accordance with other studies [36], the polymorphism T58P has a high prevalence, as evidenced by it being detected in all analyzed HCV subtype 4d sequences.

## 5. Conclusions

In conclusion, the lack of adherence to conventional healthcare facilities by the most affected people with hepatitis C, along with the emergence of RAS to antivirals used in the treatment, may prevent the achievement of Goal 3 from the United Nations’ 2030 Agenda, which aims to eliminate viral hepatitis as a Public Health Problem by 2030. The methadone program developed in mobile health units in the city of Lisbon has provided an additional opportunity to treat these chronically infected drug users, reducing the negative impact of hepatitis C on individual and public health.

Although current guidelines do not recommend screening for baseline RAS before initiating antiviral therapy, this strategy might be beneficial in the most affected vulnerable groups. In fact, it may maximize the efficacy of drugs for treatment and help disclose other factors, such as the presence of recombinant forms or mixed infections that can make hepatitis C treatment difficult. 

Even though this study presented an initial approach to investigate baseline RAS in the NS5A region only, it is also important to point out that the development of techniques for HCV resistance testing should be based on the analysis of all three regions targeted by the antivirals (NS3, NS5A, and NS5B)—with this being our future research direction. New strategies based on the use of whole genome sequencing are currently under development at the national reference laboratory. This new approach will contribute to better surveillance and control of HCV infection and also HIV-1 and HIV-2 infections, helping to support physicians in the treatment of these infections.

## Data Availability

Nucleotide sequences obtained from Sanger Sequencing were submitted to the GenBank database with the accession numbers ON506742-ON506825. The consensus sequence of the mixed infection detected using NGS was also submitted to GenBank with the accession number OP179320. NGS raw reads generated were deposited in the European Nucleotide Archive under BioProject accession no. PRJEB55165 (https://www.ebi.ac.uk/ena/browser/view/PRJEB55165; (accessed on 20 June 2022) run accession numbers ERR10028668-ERR10028751). See Supplementary Table S1.

## Supplementary Materials

The following supporting information can be downloaded at: https://www.mdpi.com/article/10.3390/pathogens12060754/s1, Figure S1: Similarity plot analysis of the recombinant form 2k/1b; Table S1: Epidemiological and clinical data from samples under study.

## References

[1] Martinez M.A., Franco S. (2021). Therapy Implications of Hepatitis C Virus Genetic Diversity. Viruses.

[2] World Health Organization Global Hepatitis Report 2017. https://www.who.int/publications/i/item/9789241565455.

[3] Simmonds P. (2013). The origin of hepatitis C virus. Curr. Top. Microbiol. Immunol..

[4] Gale M., Foy E.M. (2005). Evasion of intracellular host defence by hepatitis C virus. Nature.

[5] Smith D.B., Bukh J., Kuiken C., Muerhoff A.S., Rice C.M., Stapleton J.T., Simmonds P. Classification and Genotype/Subtype Assignments of Hepatitis C Virus. ICTV. https://ictv.global/sg_wiki/flaviviridae/hepacivirus.

[6] Dietz C., Maasoumy B. (2022). Direct-Acting Antiviral Agents for Hepatitis C Virus Infection-From Drug Discovery to Successful Implementation in Clinical Practice. Viruses.

[7] Vega S., Neira J.L., Marcuello C., Lostao A., Abian O., Velazquez-Campoy A. (2013). NS3 protease from hepatitis C virus: Biophysical studies on an intrinsically disordered protein domain. Int. J. Mol. Sci..

[8] Calleja J.L., Uerena S., Perello C., Crespo J. (2016). NS5A Resistance: Clinical implications and treatment possibilities. AIDS Rev..

[9] Sorbo M.C., Cento V., Di Maio V.C., Howe A.Y.M., Garcia F., Perno C.F., Ceccherini-Silberstein F. (2018). Hepatitis C virus drug resistance associated substitutions and their clinical relevance: Update 2018. Drug Resist Updat..

[10] Pádua E., Avó A.P., Almeida C., Água Doce I., Cortes Martins H. (2015). Conhecer a Diversidade do Vírus da Hepatite C para Além da Frequência dos Genótipos em Amostras Analisadas entre 2009 e 2014 no Laboratório de Referência do Instituto Nacional de Saúde Dr. Ricardo Jorge. Acta Med. Port..

[11] Pádua E., Avó A.P., Água Doce I., Almeida C., Martins H.C. (2014). Subtipagem do vírus da Hepatite C por sequenciação: Um contributo para o conhecimento da diversidade genética. Obs. Bol. Epidemiológico INSA.

[12] Silva M.J., Pereira C., Loureiro R., Balsa C., Lopes P., Água-Doce I., Belo E., Martins H.C., Coutinho R., Pádua E. (2017). Hepatitis C in a Mobile Low-Threshold Methadone Program. Eur. J. Gastroenterol. Hepatol..

[13] Andre-Garnier E., Besse B., Rodallec A., Ribeyrol O., Ferre V., Luco C., Le Guen L., Bourgeois N., Gournay J., Billaud E. (2017). An NS5A single optimized method to determine genotype, subtype and resistance profiles of Hepatitis C strains. PLoS ONE.

[14] Hall T.A. (1999). BIOEDIT: A user-friendly biological sequence alignment editor and analysis program for Windows 95/98/ NT. Nucleic Acids Symp Ser..

[15] Borges V., Pinheiro M., Pechirra P., Guiomar R., Gomes J.P. (2018). INSaFLU: An automated open web-based bioinformatics suite “from-reads” for influenza whole-genome-sequencing-based surveillance. Genome Med..

[16] Tamura K., Stecher G., Kumar S. (2021). MEGA11: Molecular Evolutionary Genetics Analysis Version 11. Mol. Biol. Evol..

[17] Naruya S., Masatoshi N. (1987). The neighbor-joining method: A new method for reconstructing phylogenetic trees. Mol. Biol. Evol..

[18] Kimura M. (1980). A simple method for estimating evolutionary rates of base substitutions through comparative studies of nucleotide sequences. J. Mol. Evol..

[19] Felsenstein J. (1985). Confidence Limits on Phylogenies: An Approach Using the Bootstrap. Evolution.

[20] Pawlotsky J.-M. (2016). Hepatitis C Virus Resistance to Direct-Acting Antiviral Drugs in Interferon-Free Regimens. Gastroenterology.

[21] Perales C., Chen Q., Soria M.E., Gregori J., Garcia-Cehic D., Nieto-Aponte L., Castells L., Imaz A., Llorens-Revull M., Domingo E. (2018). Baseline hepatitis C virus resistance-associated substitutions present at frequencies lower than 15% may be clinically significant. Infect Drug Resist..

[22] Christensen K.T., Pierard F., Beuselinck K., Bonsall D., Bowden R., Lagrou K., Nevens F., Schrooten Y., Simmonds P., Vandamme A.M. (2022). Full-genome next-generation sequencing of hepatitis C virus to assess the accuracy of genotyping by the commercial assay LiPA and the prevalence of resistance-associated substitutions in a Belgian cohort. J. Clin. Virol..

[23] Viazov S., Ross S.S., Kyuregyan K.K., Timm J., Neumann-Haefelin C., Isaeva O.V., Popova O.E., Dmitriev P.N., El Sharkawi F., Thimme R. (2010). Hepatitis C virus recombinants are rare even among intravenous drug users. J. Med. Virol..

[24] Del Campo J.A., Parra-Sánchez M., Figueruela B., García-Rey S., Quer J., Gregori J., Bernal S., Grande L., Palomares J.C., Romero-Gómez M. (2018). Hepatitis C virus deep sequencing for sub-genotype identification in mixed infections: A real-life experience. Int. J. Infect Dis..

[25] Pham S.T., Bull R.A., Bennett J.M., Rawlinson W.D., Dore G.J., Lloyd A.R., White P.A. (2010). Frequent multiple hepatitis C virus infections among injection drug users in a prison setting. Hepatology.

[26] Kalinina O., Norder H., Mukomolov S., Magnius L.O. (2002). A Natural Intergenotypic Recombinant of Hepatitis C Virus Identified in St. Petersburg. J. Virol..

[27] Avó A.P., Água-Doce I., Andrade A., Pádua E. (2013). Hepatitis C Virus Subtyping Based on Sequencing of the C/E1 and NS5B Genomic Regions in Comparison to a Commercially Available Line Probe Assay. J. Med. Virol..

[28] Llamosas-Falcón L., Shield K.D., Gelovany M., Hasan O.S.M., Manthey J., Monteiro M., Walsh N., Rehm J. (2021). Impact of alcohol on the progression of HCV-related liver disease: A systematic review and meta-analysis. J. Hepatol..

[29] Eltahla A.A., Leung P., Pirozyan M.R., Rodrigo C., Grebely J., Applegate T., Maher L., Luciani F., Lloyd A.R., Bull R.A. (2017). Dynamic evolution of hepatitis C virus resistance-associated substitutions in the absence of antiviral treatment. Sci. Rep..

[30] Sarrazin C. (2016). The importance of resistance to direct antiviral drugs in HCV infection in clinical practice. J. Hepatol..

[31] Papaluca T., O’Keefe J., Bowden S., Doyle J.S., Stoove M., Hellard M., Thompson A.J. (2019). Prevalence of baseline HCV NS5A resistance associated substitutions in genotype 1a, 1b and 3 infection in Australia. J. Clin. Virol..

[32] Lontok E., Harrington P., Howe A., Kieffer T., Lennerstrand J., Lenz O., McPhee F., Mo H., Parkin N., Pilot-Matias T. (2015). Hepatitis C virus drug resistance-associated substitutions: State of the art summary. Hepatology.

[33] Aissa Larousse J., Trimoulet P., Recordon Pinson P., Tauzin B., Azzouz M.M., Ben Mami N., Cheikh I., Triki H., Fleury H. (2015). Prevalence of hepatitis C virus (HCV) variants resistant to NS5A inhibitors in naïve patients infected with HCV genotype 1 in Tunisia. Virol. J..

[34] McPhee F., Hernandez D., Zhou N. (2017). Effect of Minor Populations of NS5A and NS5B Resistance-Associated Variants on HCV Genotype-3 Response to Daclatasvir plus Sofosbuvir, with or without Ribavirin. Antivir. Ther..

[35] Manns M., Samuel D., Gane E.J., Mutimer D., McCaughan G., Buti M., Prieto M., Calleja J.L., Peck-Radosavljevic M., Müllhaupt B. (2016). Ledipasvir and sofosbuvir plus ribavirin in patients with genotype 1 or 4 hepatitis C virus infection and advanced liver disease: A multicentre, open-label, randomised, phase 2 trial. Lancet Infect Dis..

[36] Schnell G., Tripathi R., Beyer J., Reisch T., Krishnan P., Dekhtyar T., Irvin M., Hall C., Yu Y., Mobashery N. (2018). Characterization of demographics and NS5A genetic diversity for hepatitis C virus genotype 4-infected patients with or without cirrhosis treated with ombitasvir/paritaprevir/ritonavir. J. Viral Hepat..

