# Supplementary material for: Looking at the Molecular Target of NS5A Inhibitors throughout a Population Highly Affected with Hepatitis C Virus
Source: Pathogens. 2023 May 24;12(6):754. doi: 10.3390/pathogens12060754 (PMC10302339; doi:10.3390/pathogens12060754)
Supplement: Supplementary file 1 [file pathogens-12-00754-s001.zip › Pathogens_supplementary _figure_S1_NS5AHCV_09_02_23.pdf]

Supplementary Figure S1

Looking at the Molecular Target of NS5A inhibitors throughout a Population Highly Affected with Hepatitis C Virus

Diogo Ramos[1], Miguel Pinto[2], Rodrigo Sousa Coutinho[3], Carolina Silva[1], Miriam Quina[1], João Paulo Gomes[2] and Elizabeth Pádua[1]*

[1] Reference Laboratory of HIV and Hepatitis B and C, Department of Infectious Diseases, National Institute of Health, Av. Padre Cruz, P-1649-016 Lisbon, Portugal

[2] Genomics and Bioinformatics Unit, Department of Infectious Diseases, National Institute of Health, Av. Padre Cruz, P-1649-016 Lisbon, Portugal

[3] Association Ares do Pinhal, Association for the Rehabilitation of Drug Addicts, Low-Threshold Methadone Substitution Program, R. José Inácio Andrade, Loja 2 – A6 – 10B Quinta do Lavrado, P-1900-418 Lisbon, Portugal

***Corresponding author:** elizabeth.padua@insa.min-saude.pt

SimPlot – Query: A61-TX178

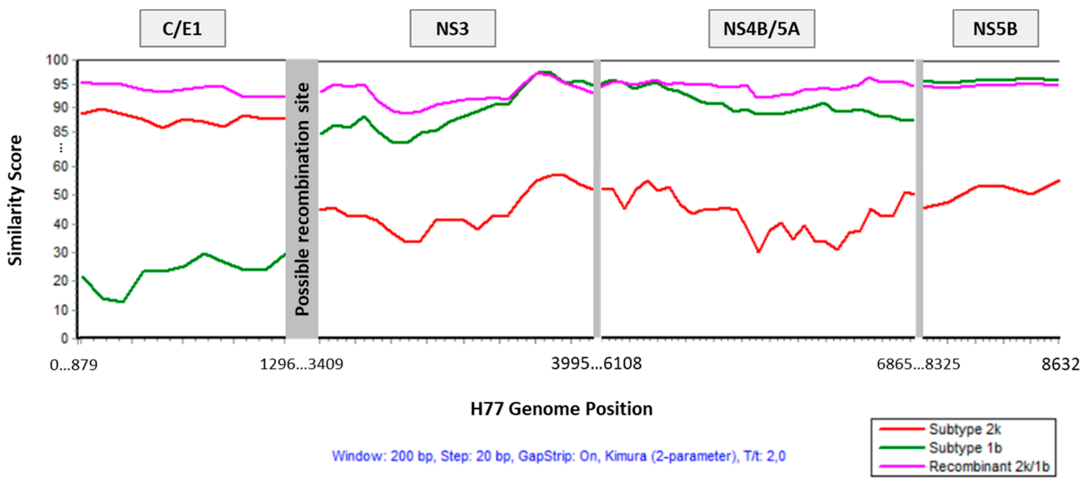

Figure S1. Similarity plot analysis of the recombinant form 2k/1b, detected in sample A61-TX178, against reference strains (**2k:** AB031663, KC197234; **1b:** EU781827, EU781828 and **2k/1b:** AY587845) based on the C/E1, NS3, NS4B/NS5A and NS5B HCV genomic regions analyzed by Sanger sequencing in the laboratory. The possible region for the recombination site is highlighted and corresponds to nucleotides 1296 to 3409 (H77 reference positions).
